# Multisensory Cues for Gait Rehabilitation with Smart Glasses: Methodology, Design, and Results of a Preliminary Pilot

**DOI:** 10.3390/s23020874

**Published:** 2023-01-12

**Authors:** Silvia Imbesi, Mattia Corzani

**Affiliations:** 1 Department of Architecture, University of Ferrara, 44121 Ferrara, Italy; 2 Department of Electrical, Electronic, and Information Engineering, University of Bologna, 40126 Bologna, Italy

**Keywords:** design methodologies, gait parameters, smart glasses, multisensory cues, mHealth, testing protocol

## Abstract

Recent advances in mobile technology have shown that augmented unisensory feedback can be leveraged to improve gait using wearable systems, but less is known about the possible benefits and usability of multisensory (i.e., multimodal) feedback. This paper introduces the preliminary results of an innovative research project aiming to develop an mHealth system including Android smart glasses, and providing multisensory cues for gait rehabilitation of people affected by Parkinson’s disease in and out of the medical context. In particular, the paper describes a preliminary pilot focusing on the design of visual, auditory, and haptic cues, and testing the design methodologies to be used in further developments of the project. Considered research questions were: Which kinds of images, sounds, and vibrations mostly influence gait speed, stride length, and cadence? Which are the ones stressing the user the least? Which ones induce the most immediate reaction? Thus, in this starting part of the research project, different typologies of sensory cues were designed, tested, and evaluated considering quantitative and qualitative parameters to properly answer the research questions.

## 1. Introduction

It is well known that in recent decades the global population, especially in the most developed countries, has become older. This scenario is significantly affecting public expenses related to older people’s healthcare, giving a burden to young generations who will have to ensure the economic, social, and welfare balance that the elderly in increasing numbers will need. It would be desirable to improve welfare expenses in research for products, services, systems, and processes for healthcare purposes, especially for prevention of age-related diseases. Promoting healthy aging, impairment prevention, and health parameter monitoring can improve quality of life not only for older people but for the whole society.

The research project related to the preliminary pilot described in this paper focuses on innovative and inclusive solutions for frail people needing medical assistance to rehabilitate their physical condition. Specifically, those considered for the final project were people with Parkinson’s disease (PD). PD is classified as a neurologic disorder causing four cardinal symptoms: tremor, rigidity, postural instability, and bradykinesia [1], which can give the person a wide range of physical impairments, including a particularly destructive effect on their physical abilities related to gait [2]. Gait problems commonly lead to disability in people with PD. PD can cause gait instability and patterning difficulty, bringing increased cadence, reduced stride length, freezing of gait, and reduced velocity. Although pharmacological therapies exist, gait impairments remain persistent and are related to a reduction of personal independence and safety, confirming the importance of developing alternative approaches and strategies for these problems’ management [3].

Physical exercise and physiotherapy have been shown to improve PD motor impairments by decelerating and postponing motor decline [4]. Moreover, exercise effects can be enhanced by increasing subjects’ cognitive engagement with practice through the provision of cueing or feedback in PD [5]. Cueing is via temporal or spatial sensory stimuli that ameliorate and facilitate repetitive movements by providing an explicit motor target. Different reviews indicate that various cueing modalities immediately affect gait and health-related quality of life [3,6,7]. Interestingly, recent advances in mobile technology have shown that augmented uni-sensory feedback can be leveraged to improve gait using wearable systems [8], but less is known about the possible benefits and usability of multisensory (i.e., multimodal) feedback.

To our knowledge, to date, we lack structured knowledge on the design of visual, audio, and haptic cueing stimuli. More often, a rhythmic sound is provided as a cue strategy using a metronome beat to induce a specific target cadence in persons with PD.

This paper introduces the preliminary results of design research analyzing how sensory stimuli can influence people’s gait. This research project started from an inter-university agreement, between the Universities of Bologna and Ferrara in Italy, for the analysis and development of smart solutions for the rehabilitation of gait and posture in people affected by Parkinson’s disease (PD).

This research aimed at designing several sensory cues and the mHealth system setup for the validation in laboratory context. Specifically, several auditory, visual, and haptic cues were tested to understand which sensory channels are most sensitive to which type of stimulation and which gait parameters are significantly influenced by this kind of stimuli, characterized by low cognitive involvement. The involvement of users in the design process aimed to improve the system usability, allowing them to take advantage of a satisfying experience that will help them perform, in a better way, specific activities of their life [9].

The system to be developed is targeted to PD subjects that are facing the initial and intermediate stages of the disease (Hoehn and Yahr: I–II), dealing with several ailments but most of all with a physical decline that affects walking and motor skills in general, impacting functional independence, well-being, and health-related quality of life [10].

Results obtained in this preliminary part of the research are supposed to be used for people with PD in ambulatory contexts and outside specialized centers to rehabilitate postural and transient gait disturbances (including Pisa syndrome and occasional, context-specific festination and freezing of gait) and to provide gait training at home, respectively.

The use of external sensory cues (e.g., auditory, visual, and haptic) to reinforce attention toward the task [11,12] is an effective gait-rehabilitation solution for persons with PD; the cues stimulate the voluntary executive component of action [13,14] by activating the attentional-executive motor control system and bypassing the dysfunctional, habitual, sensorimotor basal ganglia network [15]. In the past, auditory cueing during gait has typically been provided continuously in an open loop (regardless of gait performance) [8]. In the therapeutic context, people are instructed to match foot strike with each beat of the auditory rhythm: these so-called ‘cues’ [16] improve the movement’s initiation or continuation. Recent innovations in wearables and mobile technology provide promising new ways to offer closed-loop feedback to people with PD during gait [7], which can overcome traditional open-loop cues, providing customized cueing: stimuli are triggered in real time when gait deviates from normal, thus providing patients with instant feedback on their performance.

In the sensory feedback field, proprioceptive feedback (such as haptic stimuli) has been shown to require little or no cognitive processing or attention [17]. In addition, a multisensory approach enhances perceptual processing [18], which is reduced in PD subjects [19]. Therefore, using a different combination of sensory inputs might significantly change the effectiveness and efficiency of motor responses to the feedback. Due to technological advances, visual feedback is feasible through SG [20]. SG represents an ideal modality to offer tailored feedback and assistance to people with PD in daily living situations. Indeed, McNaney et al. [21] found that participants with PD were generally positive about SG as an everyday assistive device; however, usability issues and social stigma still hinder its general acceptance.

This project’s scope was to test and design an innovative wearable gait rehabilitation solution obtained by integrating the Vuzix Blade SG [22] into the smartphone-based CuPiD system described above [23]. Thus, the resulting gait rehabilitation solution allows therapists to design customized rehabilitation programs based on individual patient sensory preferences, cognitive aspects, and medical knowledge. In particular, Blade SG can augment reality by overlaying pertinent information on top of the user’s visual field: projecting a bi-dimensional image on the right lens. It can also trigger vibratory feedback on its right and left temples.

## 2. Materials and Methods

During the planning phase, general objectives and strategies were set. Considering the complexity of the whole project, the design process was divided into separate cycles, each one referred to a single component of the system submitting sensory cues to rehabilitate users’ gait.

In particular, authors decided to start the project with a preliminary study focusing on the design of visual, auditory, and haptic cues, and testing the methodology used in the other parts of the project. The first research questions needing response were: Which kind of images, sounds, and vibrations greatly influence gait speed, stride length, and cadence? Which are the ones stressing the user the least? Which ones induce the most immediate reaction? Thus, in this first design cycle, authors decided to design and test different kinds of sensory cues considering quantitative and qualitative parameters to properly answer the research questions.

People involved in this project as primary and secondary users belonged to the following three groups:

- Professionals belonging to the project team coming from different research fields (a designer, a biomedical engineer, and an expert in computer science);
- Stakeholders bringing interests in the project context (a neurologist, a physiotherapist, and a company producing mHealth systems);
- A group of five voluntary users to test the designed cues (range of age 32–54, not affected by significant impairments).

For the definition of the operating context of the system, the multidisciplinary team decided to consider as the primary environment the ambulatory, in the presence of a medical operator; as a secondary environment, outdoor paths selected by the user were considered.

The analyzing phase, both for human requirements and technical constraints, was developed by reviewing coherent scientific literature, analyzing existing systems operating with similar technologies and for similar users, and interviewing the different categories of users involved in the project.

The analysis of users led to considerations on motor and cognitive performance concerning the submission of sensory and multisensory cues for the empowerment and monitoring of gait performance. Considering motor dysfunctions related to PD, it is clear that personalized gait rehabilitation could ameliorate the motor performance improving gait slowness, variability, asymmetry, and even postural control [24].

Besides, from the cognitive point of view, the analysis showed that simulations should occur through cues that do not represent or pronounce the action to be performed but rather try to induce it through intuitive and straightforward communication, not requiring cognitive or interpretative processing by the user [25].

### 2.1. CuPiD System

In studies of gait problems peculiar to PD, recent advances in mobile technology have shown that augmented sensory feedback can be leveraged to improve walking [8,25]. The project described in this paper started as an innovative implementation of an existing gait rehabilitation system called CuPiD that makes persons aware of their gait performance through augmented verbal feedback [22].

The CuPiD system works using only the auditory channel by earphones; thus it would be desirable to enrich the system with more types of cues involving additional senses. The CuPiD system is given as a project invariant; it was chosen to associate it with Smart Glasses (SG), able to submit other kinds of sensory cues.

Thanks to SG, real-time visual and haptic feedback are also possible: this research project investigated strategies to design an mHealth system obtained by integrating the Android-based CuPiD system with a commercial Android-based SG (Vuzix Blade) [23].

The CuPiD system consists of a smartphone Android app that processes data streamed from the IMUs positioned on the shoes, and elaborates gait parameters to trigger real-time verbal feedback on gait. Innovatively, authors modified the CuPiD-smartphone app to allow information exchange via Bluetooth with SG. These can augment reality by overlaying pertinent information on top of the user’s visual field: projecting a bi-dimensional image on the right lens, the display user interface. It also can trigger dual haptic/vibratory feedback on its right and left temples. Thus, when the CuPiD-smartphone app triggers real-time feedback, the user can receive the SGs’ multisensory cues without any action needed.

To date, there are no similar mHealth systems that provide real-time multisensory cues for gait rehabilitation in the three different human sensory channels. Recent advances in mobile technology have shown that augmented unisensory feedback can be leveraged to improve gait using wearable systems, but less is known about the possible benefits and usability of multisensory feedback. This project aimed to develop an mHealth system which includes an Android SG to provide and test multisensory cues in gait rehabilitation.

To quantitatively analyze our results, we adopted the mGait system (mHealth Technologies SRL, Bologna, Italy), a CE-marked medical device, to evaluate the spatiotemporal gait parameters [26]. The mGait system was used with two inertial sensors worn on the feet, and data were transmitted via Bluetooth to the mGait smartphone app.

Several spatiotemporal gait parameters were identified as significant for gait assessment [11]: gait speed, stride length, swing time variability, cadence variability, stance time variability, gait speed variability, stride length variability, cadence, swing time, stance time, cadence asymmetry, swing time asymmetry, stance time asymmetry, and stride length asymmetry. Therefore, these parameters were selected as benchmarks for comparing the different performances obtained during the trials with users.

### 2.2. Design of Sensory Cues

During the creating phase, several design solutions were developed to fulfil human requirements obtained from scientific literature and from the interaction with representatives of the selected categories of users (medical operators, technical operators and final users).

#### 2.2.1. Visual Cues

Visual cues have been developed to give the user a suggestion on the cadence to be adopted, using images not requiring interpretation but giving a rhythmic cadence to follow without distracting the user from the visual focus of the performing path.

Moreover, it was chosen to avoid complex writing and icons, preferring simple figures that do not imply interpretation in reading. Among the simple analyzed figures, the circle was considered particularly immediate, and thus re-proposed in several variations.

The visual patterns have all been developed according to a basic module of two “beats” (right and left step), to be repeated in a loop for the duration deemed appropriate, according to the pre-established frequency (in the present experiment 100 bpm).

After experimenting with the SG display using different color palettes (in closed and open environments and other lighting conditions), the colors chosen were white and purple in various degrees of saturation. The selected colors were the most visible in different environmental conditions and seemed to be the most faithfully reproduced compared to the original image.

Concerning visualization, it was chosen to opt for two solutions: patterns composed of two figures, projected in correspondence with the right and left step; and patterns consisting of six figures which, compatible with the reproduction capabilities of the device, are supposed to create a sort of animation in which the right step corresponds to frame 1, and the left step to frame 4.

Regarding the number and arrangement of the figures represented in the images to be projected, the three compositional solutions elaborated were a single central figure, a single tilting figure, and a double tilting figure.

Developed visual cues were called A1, A2, A3, A4, A5, A6 (Supplementary Materials).

#### 2.2.2. Auditory Cues

Even sound patterns, like visual ones, can suggest a predetermined cadence to which to adapt the pace. In creating the cues belonging to this typology, authors chose to prefer rhythmic sounds that stressed the user as little as possible. The rhythms of the metronome or sounds that can be intuitively connected to the world of robotics were limited due to the feelings of anxiety and burden they transmit, opting for families of sounds closer to the human or musical dimension. Authors also chose to exclude the reproduction of a single sound, considered more annoying and agitated, in favor of some different sounds whose rhythmic alternation aims to suggest a postural alternation and a combined physical movement that can evoke the right and left step.

Developed auditory cues were called B1, B2, B3, B4, B5, B6 (Supplementary Materials).

#### 2.2.3. Haptic Cues

SG can exercise vibratory cues on the support point of both the right and left temples. Among the three signals, vibration is the one that presents less possibility of variation, being monotonic and programmable in the duration variable only. In the haptic cues, a frequency of 100 Hz was adopted according to the pre-established frequency of 100 bpm for the cadence.

Vibratory cues’ variants have been developed aiming, as with the previous signals, to invite the user to a regular pace. The basic principle is to suggest a rhythm with right and left vibration corresponding to the impact of the right and left foot during walking. The temples vibrated singularly and separately, each one corresponding to the step on its side.

Developed haptic cues were called C1, C2 (Supplementary Materials).

#### 2.2.4. Multisensory Cues

Sensory cues previously developed were combined using two or three different channels per time, to create a new category of cues stimulating more senses at the same time.

Developed multisensory cues were named D1, D2, D3. (Supplementary Materials).

### 2.3. Collection of Qualitative and Quantitative Information

During the verifying phase, qualitative information was obtained by a short questionnaire submitted to the user while testing the cues. In addition, quantitative information was obtained from gait data monitored during the trial.

This information was elaborated and interpreted to obtain evaluations and to fulfil users’ needs for simultaneous comparison of factors influencing users’ gait.

Authors want to specify that in this design cycle, five users were tested. It was chosen to involve in tests users not belonging to a specific category or reporting particular health issues. The main reason was that, from the pandemic perspective, we did not want to expose frail people to potential health risks. Another reason was that in this first part of the experimentation, not explicitly related to pathological behaviors, it was possible to consider a human-centered approach considering the broad human perception instead of that of PD sufferers. As specified before, users actually involved in the pilot did not declare specific impairments and belonged to a range compatible with the onset of the first symptoms of early PD.

#### 2.3.1. Interview

The individual interview reported in Table 1 was divided into two parts: the first one was focused on submitted sensory cues, asking for scores on a scale of 1–5, while the second one consisted of an open questionnaire about the use of SG (Table 1).

#### 2.3.2. Testing Protocol

Regarding the collection of quantitative data, a specific non-clinical testing protocol was applied to monitor several parameters related to the gait, beyond the medical ambulatory. Operators collected user data such as age, height, sex, and dominant foot to understand the performances of different people. Everything was filmed to have a gold standard for verifying doubts about testing (framing both the walking subject and the operator sending the stimulus).

During the tests, every user was equipped with the mGait system and asked to walk along a straight path. The operator sent the sensory cues via an ad hoc smartphone app (to mimic the operation of the CuPiD system). All cues were set on the cadence of 100 steps per minute, a value close to the average rhythm suggested by the literature [27].

Before carrying out the tests with sensory cues, a walk was performed along the same path, but without the induction of signals, to take these test parameters as a reference for the following ones. Even if the suggested cadence was not targeted to the specific user, it did not seem to be a cause for discouragement; however, it allowed a comparison of the responses to single signals.

The user was invited to walk the path using their usual pace; between 5 and 7 m from the start of the walk (range depended on the basic step length of the tested user, so the tests of different users could be comparable in duration and number of steps), the cue was transmitted; it was then interrupted only at the end of the path (the subject usually walked at least 15 m after receiving the signal).

**Figure 1:**
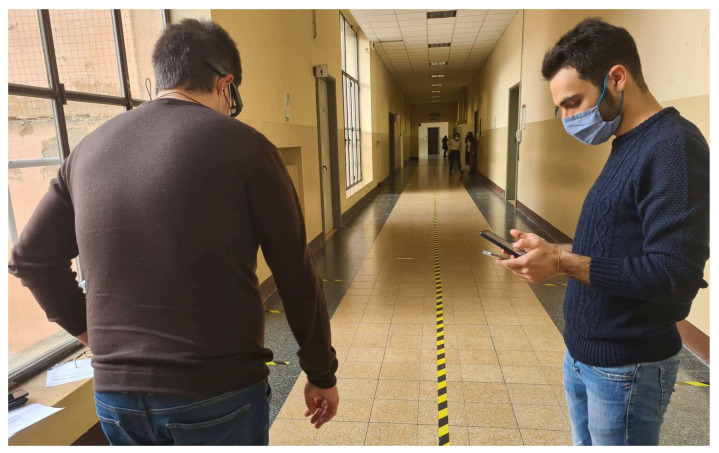
A user walks along the selected path while receiving a sensory cue.

In the submission of the stimulation, attention was paid to the correct synchronization between the incoming signal and the cadence of the walk: the signal was sent remotely by the researcher, triggering the cues to the subject while the right heel touched the floor, and the step was noted (Figure 1).

After the first baseline test, the user was invited to align with the cadence suggested by the mono or multisensory stimuli transmitted by operators.

In total, each subject performed 18 tests following this scheme:

1 baseline trial without stimuli as a reference;

14 monosensory tests and 3 multisensory tests with total randomized administration order (A1:A6, B1:B6, C1:C2, D1:D3).

### 2.4. Qualitative and Quantitative Evaluations

At this point of the design process, the authors’ aim was to experiment with a strategy to combine obtained results to hierarchize the cues’ effectiveness, considering all detected parameters and users’ points of view, comparing them, and weighing their relative and absolute importance. This hierarchy was supposed to allow the selection of a small number of sensory cues to be submitted to people with PD in the following steps of the research project, avoiding wasting time in testing ineffective solutions. The second aim of this initial project phase, slowed down by pandemic restrictions, was to test an innovative methodology able to match qualitative and quantitative aspects for exhaustive evaluation of the developed solutions.

Therefore, it was decided to use the Quality Function Deployment (QFD) tool to manage the complexity of collected data and try to make a qualitative and quantitative evaluation of the results obtained in the verifying phase.

QFD is a design tool traditionally used to manage complexity in design processes within industrial companies, including sectors involved in the product’s development [28].

The traditional QFD tools express relationships between the needs and characteristics of the product or system to be developed [29,30], to balance them in the needs satisfaction level assessment. It consists in a matrix reporting in the lines the needs expressed by different categories of users previously interviewed (final users, installer, buyer, representatives of the different sectors of the company, etc.), while in columns are reported the measurable characteristics of the product to be designed. When the matrix is filled with needs and characteristics, involved users are firstly asked to express a mark from 1 to 5 indicating the needs importance. Then, they will express a degree of correlation evaluating how different values of the characteristic could impact on the need satisfaction. The combination of needs importance and correlation degree will generate in the lower part of the matrix values representing characteristics’ absolute and relative importance. The obtained results allow evaluation of which characteristics of a product need to be improved to better satisfy users’ needs; to understand which needs are not satisfied from current characteristics; and to discover which characteristics should not be considered for users’ satisfaction, etc.

However, due to the results of the previously described analysis, authors decided to experiment with an innovative use of the QFD tool, considering designed sensory cues instead of the project characteristics. The combination of users’ needs and cues prototypes showed which signals prompted the best performances considering different qualitative and quantitative aspects. Specifically, the QFD tool suggested a classification of the cues mixing users’ suggestions with collected data, helping the multidisciplinary team in the interpretation and comparison of monitored performances.

#### Parameters

Qualitative parameters collected and analyzed were levels of perceived invasiveness and annoyance, and perceived difficulty in following the signal. The quantitative ones were the difference between users’ cadence and the target one, the number of steps to reach the cadence of 100 steps/min, the users’ cadence variability, the rhythmic asymmetry (users’ asymmetry index on cadence, and rhythmic asymmetry), and users’ asymmetry index on cadence.

Moreover, considering that parameters were collected in tables of values difficult to quickly read by the multidisciplinary team, special attention was given to the results’ visualization, to make values intuitively accessible even to professionals without a specific background in that science field.

## 3. Results

(1) Cues’ Perceived Invasiveness and Annoyance

Users were asked, after every single test, to answer the question: “How invasive and annoying was this cue?”. They assigned every cue a score from 1 to 5 (1 not invasive, 5 very invasive).

**Figure 2:**
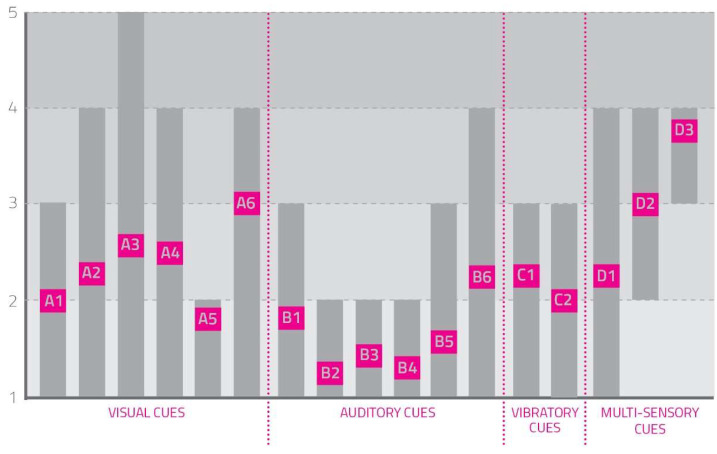
How invasive and annoying were the cues? The rectangles in the figure represent the answers given by users, their median values (the pink squares containing the name of the single cue), and distribution and variability (the length and placement of the gray rectangle).

Figure 2 represents collected values.

From this information it is possible to deduce that visual cues were the most annoying for the user, auditory cues were less disturbing depending on the specific sounds, and vibratory cues were between the previous ones. Multisensory cues seemed to be very annoying due to the number of involved senses.

Even the users’ answer variance was analyzed to understand if they all perceived cues as invasive and annoying in the same way. From the scheme it is possible to note that some cues, especially auditory ones, were similarly perceived in all tests (A1, A5, B1, B2, B3, B4, B5, C1, C2, D2, D3); for some others, particularly for visual cues, the users’ scores were both low and high (A2, A3, A4, A6, B6, D1), highlighting the influence of personal preferences and attitudes in the perception of invasiveness.

(2) Perceived Difficulty in Following the Cue Signals

Users were asked, after each test, to answer the question: “How difficult was it to follow the rhythm suggested by this cue?”. They assigned every cue a score from 1 to 5 (1 not invasive, 5 very invasive) as represented in Table 1.

**Figure 3:**
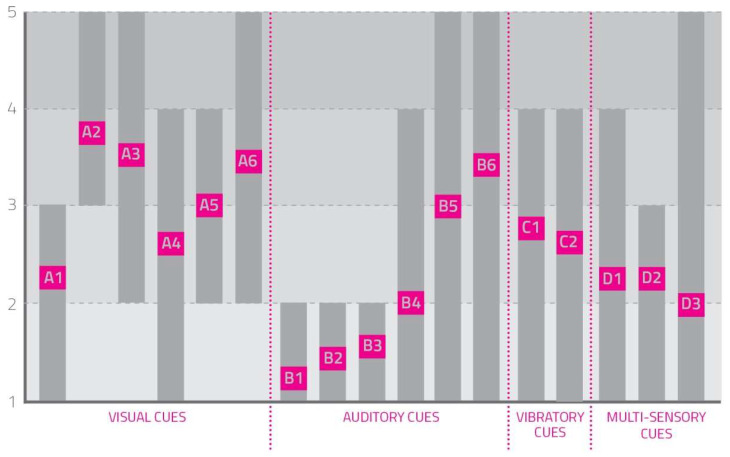
How difficult it was to follow the rhythm suggested by each cue? The rectangles in the figure represent the answers given by users, their median values (the pink squares containing the name of the single cue), and distribution and variability (the length and placement of the gray rectangle).

Figure 3 represents collected values.

From this visualization, it was possible to deduce that visual cues and some of the auditory ones were the most difficult to follow for the users; the remaining visual cues and the vibratory and multisensory ones were all perceived as moderately complex. The metronome auditory cue was the only one considered moderately challenging to follow by the users.

The scheme also analyzed the variance of answers given by users to understand if they all perceived cues as difficult to follow in the same way. It was then possible to note that only some visual and auditory cues were similarly perceived in all tests (A1, A2, A5, B1, B2, B3, D2); the other cues elicited a broad difference of given marks (A3, A4, A6, B4, B5, B6, C1, C2, D1, D3), highlighting the influence of personal attitudes in the perception of the difficulty of following the suggested rhythm.

(3) Difference between Users’ Cadence and the Target One

The system collected cadence median values from the signal onwards obtained by making the difference between users’ cadence and the target of 100 steps/min, aiming to evaluate the deviation from the target cadence (Appendix A).

**Figure 4:**
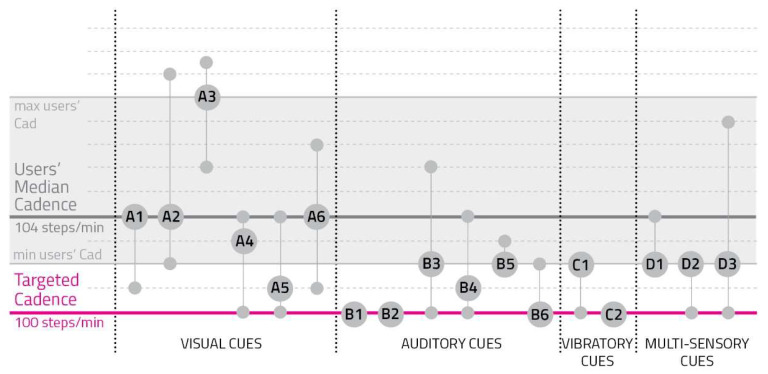
Users’ cadence during each signal. The gray section represents users’ cadences from the minimum to the maximum one. The gray bold line represents users’ median cadence, while the pink one stands for the targeted cadence. Every cue prototype corresponds to the vertical line connecting three circles: the one on the lower part of the line represents the user with the lower difference between the targeted cadence and the one shown during the test; the second circle is bigger and shows the cue code, standing for the average user’s cadence; the upper small circle indicates the user with the biggest difference between the targeted cadence and the one performed during the test.

Figure 4 represents collected values.

Figure 4 shows that auditory and vibratory cues were the most effective because they were the closest to the target cadence of 100 steps/min and produced the lowest variability among users, particularly for B1, B2, B6, and C2.

(4) Number of Steps to Reach the Cadence of 100 Steps/Min

The measured parameter was the number of steps needed to hold the users’ cadence within a value of 95–105 steps/min (5% of the target cadence) for at least 5 steps (Appendix A). The table of values shows the first step of the 5 consecutive steps. In cases where the user could not reach the alignment during the test, a value of 30 steps was reported, consistent with the maximum number of steps taken after the signal.

**Figure 5:**
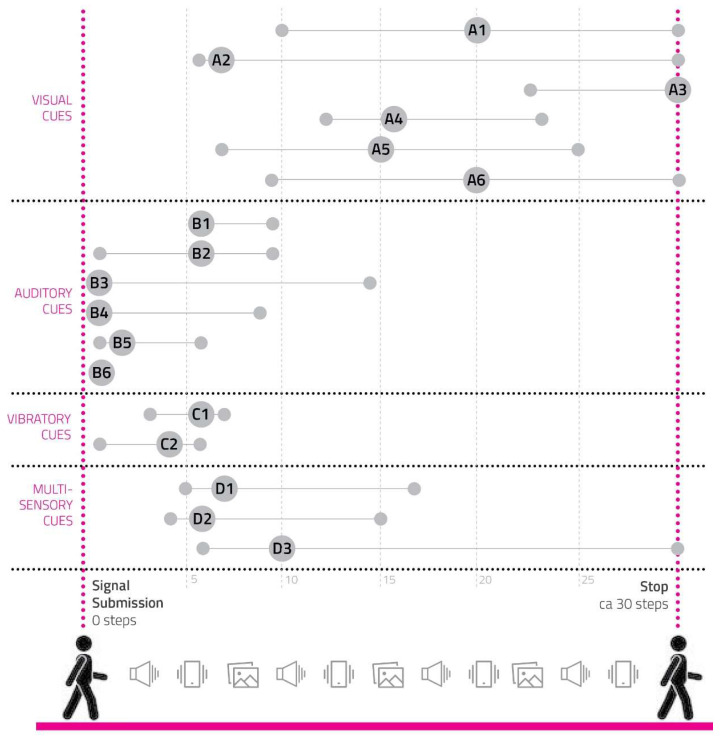
A man silhouette is represented while walking for thirty steps. The different categories of sensory cues are listed on the left, each one including several prototypes of sensory. Every cue prototype corresponds to a line connecting three circles: the first one on the left represents the user reaching the target cadence with the lowest number of steps; the second circle is bigger and shows the cue code, standing for the average number of steps users needed to reach the cadence; the last small circle on the right indicates the user needing the highest number of steps to reach the suggested cadence.

Figure 5 represents collected values.

Auditory and vibratory cues were demonstrated to be the most effective ones; they helped users to quickly align with the target cadence of 100 steps/min and with the lowest variability, particularly for B4, B5, B6, and C2.

(5) Users’ Cadence Variability

To analyze cadence variability, we extracted users’ standard deviation (Appendix A). To normalize data among subjects, all values were reported as the difference from the standard deviation obtained in the reference test. For example, a negative outcome corresponds to a lower standard deviation compared to the reference, therefore a more regular behavior.

**Figure 6:**
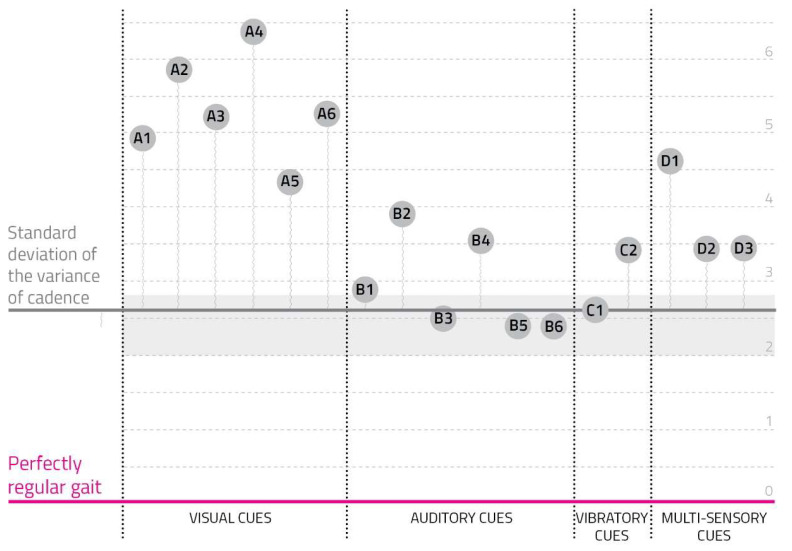
Users’ cadence variability. The gray section represents users’ standard deviation of the variance of cadence. Every cue prototype corresponds to a vertical line starting from the standard deviation of the user and ending with a circle where the cue obtained the furthest value.

Figure 6 represents collected values.

Auditory and vibratory cues led to a more constant behavior than visual ones, so their standard deviation’s variability was lower than the reference.

(6) Rhythmic Asymmetry: Users’ Asymmetry Index on Cadence

During the testing protocol, the variation between the average right and left cadence was confirmed. To normalize data among subjects, all values were reported as differences from the cadence asymmetry index obtained in the reference test (Appendix A). For example, a negative outcome corresponded to a more symmetric cadence compared to the reference, therefore a more regular rhythmic behavior.

**Figure 7:**
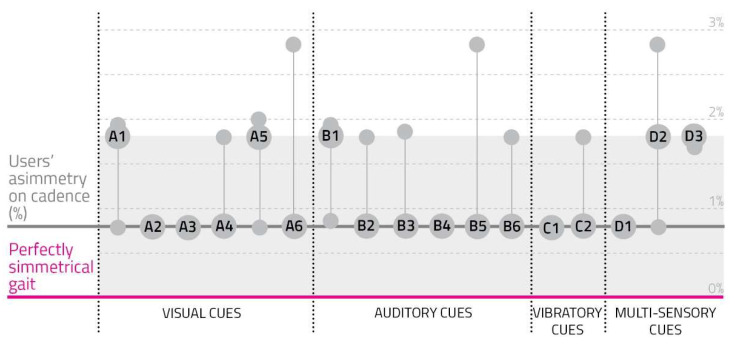
Users’ asymmetry in right and left cadence variability. The gray section represents the user’s typical asymmetry of cadence, while the pink line is a perfectly symmetrical gait. Every cue prototype corresponds to the vertical line connecting three circles: the one on the lower part of the line represents the user with the lower asymmetry on cadence; the second circle is bigger and shows the cue code, standing for the average user’s asymmetry in right and left cadence variability; the upper small circle indicates the user with the most asymmetrical gait cadence.

Figure 7 represents collected values.

A common behavior was not clear due to the high variability shown between users across all the different cue modalities.

(7) Spatial Asymmetry: Users’ Asymmetry Index on Stride Length

Percentage variations between the average right and left stride length were obtained during the test (Appendix A). To normalize data among the subjects, except from the first row, all values were reported as differences from the stride length asymmetry index obtained in the reference test. For example, a negative outcome corresponded to a more symmetric stride length compared to the reference, therefore a more regular spatial behavior.

**Figure 8:**
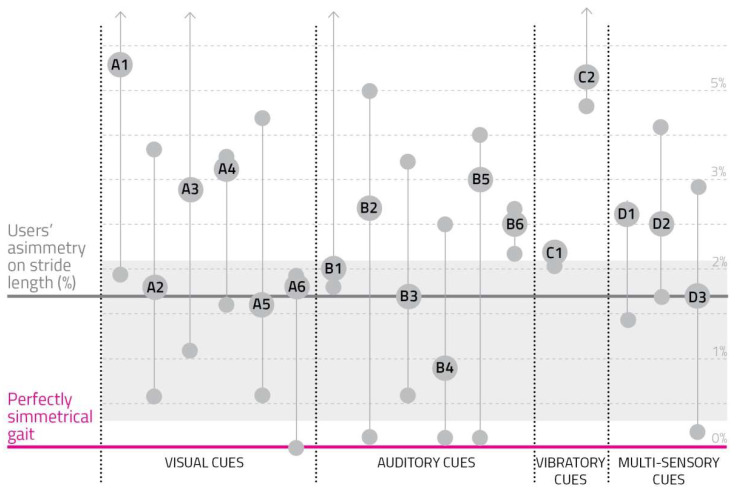
Users’ asymmetry index on stride length. The gray section represents the user’s typical asymmetry in stride length, while the pink line is a perfectly symmetrical gait. Every cue prototype corresponds to the vertical line connecting three circles: the one on the lower part of the line represents the user with the lower asymmetry; the second circle is bigger and shows the cue code, standing for the average user’s asymmetry in stride length; the upper small circle indicates the user with the most asymmetrical gait cadence.

Figure 8 represents collected values.

This result shows a general high asymmetry on stride length (up to 5%), which may be explained by the users’ attempt to modify their gait behavior to be as close as possible to the suggested cues.

### Quality Function Deployment Matrix

Once all collected quantitative and qualitative parameters were analyzed by the multidisciplinary team, users’ needs were obtained and listed in the following table (Table 2). For every need a code was assigned, the typology of the user expressing it was made explicit, and the related parameter was declared.

It needs to be specified that during the gait monitoring, values of more parameters were collected than the ones reported in this table. The multidisciplinary team selected the ones associated with needs expressed by people with PD (US) and technical operators (TO) who would use the system to train PD patients’ gait and posture.

As previously explained, obtained needs were used to fill QFD matrix lines, while columns reported all the cues tested at the beginning of the verifying phase.

All the selected needs were classified with a need number and ordered starting from the qualitative ones and following with the quantitative ones. Furthermore, a score about absolute importance was assigned to each need. As in the traditional QFD version applied by companies [30], the multidisciplinary team gave a mark from 1 to 5, expressing an opinion about every single need’s importance, concerning the whole project’s aims and scopes.

Thus, using these marks, the QFD algorithm elaborated percentage values identifying the relative importance of each need in consideration of the other ones listed in the matrix.

**Figure 9:**
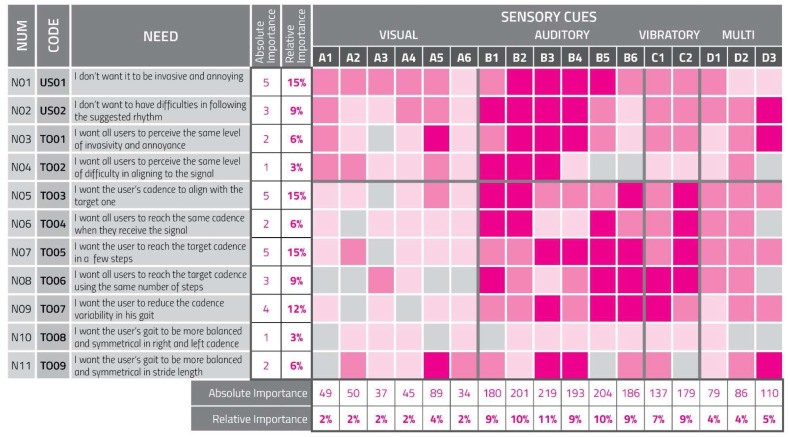
Innovative use of the Quality Function Deployment matrix as a tool to determine the most effective sensory feedback submitted to the users.

At this point of the process, considering the traditional use of the QFD matrix, the team was supposed to give a score expressing how much every cue could influence the user’s gait satisfying each specific need. Otherwise, in this specific case, scores were deducted from the values of the cue performances in every single parameter. Specifically, to determine the degree of correlation, we considered the value obtained by the considered cue for the parameter generating the need (when the cue prompted a bad performance it was considered a low level of need satisfaction and vice versa) (Figure 9).

Those scores were reported in boxes of intersection between lines and columns using the following classification:

- 0 (light gray): The cue does not respond to the need;
- 1 (light pink): The cue weakly responds to the need;
- 3 (pink): The cue satisfactorily responds to the need;
- 9 (magenta): The cue strongly responds to the need.

The additional use of colors helped comprehension, even to team members who were not experts in using and interpreting the QFD matrix [31].

In the last two lines of the matrix are reported results elaborated by QFD algorithms using absolute and relative importance of needs, combined with marks given to the degree of satisfaction cues were able to provide. The line of absolute importance shows values indicating how much every cue was generally effective in its global aim, considering the importance of every analyzed qualitative and quantitative factor. The last line showing relative importance as a percentage allows an agile reading and comparison of results obtained by the different sensory cues to satisfy users’ needs [30].

From correlation degrees between needs and cues expressed in the QFD, we obtained a hierarchy of the cues taking into consideration and combining correctly all the data collected before: B3, B2, B5, B1, B4, C2, C1, D3, A5, D1, D2, A1, A2, A3, A4, A6.

## 4. Discussion

Referring to scientific literature about multisensory feedback for gait analysis and training, different cueing modalities have a differential effect on gait rehabilitation: while visual cueing primarily improves spatial gait parameters (e.g., stride length), auditory and somatosensory cueing primarily improve temporal gait features (e.g., gait speed and cadence) [3,32]. Our outcomes align with these indications: looking at the QFD results, all the auditory and vibratory feedback reached a higher percentage than the visual feedback.

Analyzing the classification expressed by the QFD matrix, it was evident that cues remained grouped in homogeneous sensory categories, even if they were singularly evaluated.

Interestingly, among the best cues for the auditory channel, the classic metronome (B1) achieved 9% relative importance, the lowest percentage. The best one, with 11%, was the melody composed of musical instruments and electronic sounds (B3). Compared to the classic metronome, the users perceived it as less annoying, and they were able to reach the target cadence imposed by the rhythm with fewer steps. On the other hand, cues using traditional musical instruments were perceived as less bothersome and more relaxing due to their affinity to natural human sounds.

Vibratory cues were a little less high-performing than auditory ones; it was possible to compare them to the metronome. On the other hand, the possibility to transmit this signal only by the SG lenses seemed to limit significantly the potential of feedback using this sensory channel.

Visual cues obtained the lowest scores, and the reason could be that this kind of signal is suitable for issues related to space perception but is not appropriate for the submission of temporal indications to be followed, such as the cadence. It was noted that their submission forced the user to concentrate their view both on the signal and on the path. This operation could be perceived as quite confusing and could moderately compromise the user’s performance. Considering visual cues’ ability to influence movements’ spatiality rather than temporality (as auditory and somatosensory cues), authors considered to apply them in delivering punctual messages instead of suggesting a rhythm to follow for a significant amount of steps.

Nevertheless, compared to the baseline trial, all the cues tested appeared to induce a more symmetrical gait and a reduction in gait variability, confirming the positive role of cueing strategy.

Due to these results, authors decided to maintain for the following experimentation auditory and vibratory cues gaining the best results in this trial; conversely, visual and multisensory cues were supposed to be redesigned in the light of the points of weakness and strength resulting from the analysis and used for spatial purposes.

In addition, our outcomes indicated that the unimodal feedback was more effective than the multimodal one that sometimes was perceived as bothersome and confusing. Unimodal cues’ prowess could be partially linked to the choice of a simple walking paradigm, in which the subject had to walk on a linear path without obstacles, trying to follow the suggested cadence. Otherwise, in a complex motor task, multimodal feedback could be applied to exploit the specific advantages of each modality, such as the aptitude of visualizations to display alerts or spatial aspects and of sound or haptic feedback to display temporal aspects [32].

The system’s overall functioning, its tested usability, and effectiveness demonstrated by most of the cues made it possible to assess the achievement of TRL 5, as planned at the beginning of the research project.

Another strength of this new mHealth system is the common Android-based platform that allows agile and real-time communication between the SG app and the CuPiD system.

### Future Developments

From this first design cycle, according to detected parameters, authors identified some critical points such as the synchronization between the submission of the signal and the person’s gait phase; the gap between the user’s cadence and the one proposed by the system; and the location of the haptic cue limited to the right or left SG’s temples.

Another limitation was the extent to which each subject was visual-, auditory-, or somatosensory-dependent: the nervous system uses augmented sensory information differently depending primarily on individual proclivities to rely on one sensory channel over another to control motor behavior.

To overcome some of these limitations, the target cadence should be subject-specific in the following testing phase. In particular, the target cadence should be based on the one detected during a user’s reference gait during gait training. Alternatively, to comply with the therapeutic needs, the target cadence should be a modified version of the one recorded during the user’s reference gait test.

Moreover, to make our walking paradigm more challenging and thus evaluate the different characteristics of each sensory channel, in the next trial, critical aspects should be added during gait testing, such as turning or dual-tasking activity.

Finally, questionnaires or automated assessment methods should be used to evaluate patients’ sensory preferences to empower their performance due to their abilities and attitudes [33].

## 5. Conclusions

This paper presents a preliminary pilot testing sensory cues’ performances in rehabilitating the gait. Several cues were tested and data related to gait parameters were collected, elaborated, and interpreted by the multidisciplinary team. Finally, the QFD tool was applied to manage the information complexity and allow considerations about positive and negative aspects of the developed design solutions.

Results of this preliminary design study are satisfactory and encouraging for the next phases of the project. In particular, the results of the QFD tool confirm the role of sensory cues in gait rehabilitation: auditory and haptic cues reached a higher efficacy than the visual ones; while visual cues improved spatial gait parameters (e.g., stride length), auditory and somatosensory stimuli improved temporal gait features (e.g., cadence).

Moreover, a special focus was dedicated to the communication, explication, and representation of technical aspects and concepts in order to allow a deeper understanding of ongoing results of the design process to each member of the multidisciplinary research team. The representation of data, tables and functions was satisfactory and improved the involvement of researchers in the project.

Besides, the experimented methodology, especially the verifying phase, has proven to reduce the complexity of multidisciplinary design processes involving human and technological factors challenging to compare. It led to satisfactory results and proved to be particularly suitable for multidisciplinary design processes involving both human and technological issues concerning the development of smart systems targeted to niche users. This approach seems to be applicable in other kinds of research projects addressed to users with peculiar needs, giving an innovative contribution as a simplification tool to collect and combine data to evaluate and choose effective and usable design solutions.

## Figures and Tables

**Table 1:** Users individual interview.

Issue	Question	Answer
Sensory cues	Was it unpleasant to receive this signal?	Score from 1 to 5
	How hard was it to match the pace?	
Smart Glasses	How do you feel wearing smart glasses?	Open answer
	Is there anything that makes you uncomfortable?	
	How long do you think you could wear smart glasses?	
	Where would you imagine using smart glasses?	
	How do you think your walk has changed after receiving a signal?	

**Table 2:** Needs for the QFD matrix.

Num	Code	Need	User	Typology	Parameter
**N01**	**US01**	I do not want it to be invasive and annoying	Test User	Qualitative	Level of invasiveness and annoyance
**N02**	**TO01**	I want all users to perceive the same level of invasiveness and annoyance	Technical operator	Qualitative	Level of invasiveness and annoyance
**N03**	**US02**	I do not want to have difficulties in following the suggested rhythm	Test User	Qualitative	Level of difficulty in following the signal
**N04**	**TO02**	I want all users to perceive the same level of difficulty in aligning to the signal	Technical operator	Qualitative	Level of difficulty in following the signal
**N05**	**TO03**	I want the user’s cadence to align with the target one	Technical operator	Quantitative	Difference between users’ cadence and the target one
**N06**	**TO04**	I want all users to reach the same cadence when they receive the signal	Technical operator	Quantitative	Difference between users’ cadence and the target one
**N07**	**TO05**	I want the user to reach the target cadence in a few steps	Technical operator	Quantitative	Number of steps to align to the rhythm
**N08**	**TO06**	I want all users to reach the target cadence using the same number of steps	Technical operator	Quantitative	Number of steps to align to the rhythm
**N09**	**TO07**	I want the user to reduce the cadence variability in his gait	Technical operator	Quantitative	Cadence variability
**N10**	**TO08**	I want the user’s gait to be more balanced and symmetrical in right and left cadence	Technical operator	Quantitative	Asymmetry index on cadence
**N11**	**TO09**	I want the user’s gait to be more balanced and symmetrical in stride length	Technical operator	Quantitative	Asymmetry index on stride length

## Data Availability

Not applicable.

## Supplementary Materials

The following supporting information can be downloaded at: https://www.mdpi.com/article/10.3390/s23020874/s1, Video of visual cues: A1, A2, A3, A4, A5, A6; video of auditory cues: B1, B2, B3, B4, B5, B6; video of vibratory cues: C1, C2; video of multisensory cues: D1, D2, D3.

## Appendix A

In this section are reported tables about data cited in Section 2.4.

**Table A1:** Cues perceived annoyance and invasiveness.

	U1	U2	U3	U4	U5	TOT	TOT %	Variance	Average
**A1**	1	2	2	3	2	10	40%	2	2
**A2**	1	2	2	2	4	11	44%	3	2.2
**A3**	nc	2	5	1	2	10	50%	4	2.5
**A4**	1	2	2	3	4	12	48%	3	2.4
**A5**	1	2	2	2	2	9	36%	1	1.8
**A6**	1	2	4	3	3	13	52%	3	2.6
**B1**	1	3	1	2	2	9	36%	2	1.8
**B2**	1	1	1	2	1	6	24%	1	1.2
**B3**	1	2	1	2	1	7	28%	1	1.4
**B4**	error	2	1	1	1	5	25%	4	1.25
**B5**	1	1	1	2	3	8	32%	2	1.6
**B6**	1	4	1	3	2	11	44%	3	2.2
**C1**	3	2	1	2	3	11	44%	2	2.2
**C2**	3	1	1	1	4	10	40%	3	2
**D1**	1	3	1	4	2	11	44%	3	2.2
**D2**	3	3	4	2	3	15	60%	2	3
**D3**	3	4	4	4	4	19	76%	1	3.8

**Table A2:** Cues perceived difficulty in following the signal.

	U1	U2	U3	U4	U5	TOT	TOT %	Variance	Average
**A1**	1	3	2	2	3	11	44%	2	2.2
**A2**	3	4	3	4	5	19	76%	2	3.8
**A3**	nc	2	5	3	4	14	70%	3	3.5
**A4**	2	4	3	1	3	13	52%	3	2.6
**A5**	3	3	3	2	4	15	60%	2	3
**A6**	2	2	5	3	5	17	68%	3	3.4
**B1**	1	1	1	1	2	6	24%	1	1.2
**B2**	1	2	2	1	1	7	28%	1	1.4
**B3**	2	2	1	2	1	8	32%	1	1.6
**B4**	error	4	1	2	1	8	40%	3	2
**B5**	2	5	1	4	3	15	60%	4	3
**B6**	3	5	1	5	3	17	68%	4	3.4
**C1**	1	1	1	4	2	9	36%	3	1.8
**C2**	1	1	1	1	4	8	32%	3	1.6
**D1**	1	4	1	3	2	11	44%	3	2.2
**D2**	1	2	1	4	3	11	44%	3	2.2
**D3**	1	2	1	1	5	10	40%	4	2

**Table A3:** Difference between the user cadence and the target one.

	USER 1	USER 2	USER 3	USER 4	USER 5	Median	Q1	Q3
***REF***	*24* *[22;24]*	*2* *[0;2]*	*9* *[8,10]*	4 *[2;4]*	*0* *[0;2]*	***4***	*2*	*9*
**A1**	1 [−2;4]	8 [−10;−6]	4 [1,5]	4 [4;6]	0 [−2;2]	**4**	1	4
**A2**	4 [−1;10]	10 [−10,−7]	2 [0,2]	2 [−7;−1]	12 [−19;−8]	**4**	2	10
***A3***	NaN -	10 [−14;−6]	12 [8,15]	8 [−10;−6]	0 [−2;2]	**9**	6	10.5
***A4***	*3* [−4;6]	14 [−18;−8]	4 [0,14]	0 [−1;2]	0 [−2;2]	**3**	0	4
**A5**	*18* [16;20]	4 [−8,−3]	1 [−2;3]	0 [−7;4]	0 [−4;10]	**1**	0	4
**A6**	10 [8;12]	7 [−10;−4]	1 [−6;16]	4 [−4;−2]	0 [−2;6]	**4**	1	7
**B1**	0 *[0;2]*	*8* [−8;−6]	0 [0;2]	0 [0;2]	0 [−2;4]	**0**	0	0
**B2**	0 [−2;2]	12 [−14;−6]	0 [−2;4]	0 [−1;2]	0 [−2;0]	**0**	0	0
**B3**	18 [18;20]	6 [−6;−4]	0 [0;2]	0 [−2;0]	2 [−4;0]	**2**	0	6
**B4**	NaN -	10 [−12;−8]	2 [0,2]	0 [0;2]	0 [−2;0]	**1**	0	4
**B5**	14 [13;16]	3 [−4;−2]	2 [0,2]	2 [−2;0]	0 [−2;0]	**2**	2	3
**B6**	10 [8;12]	2 [−4;−2]	0 [−2;0]	0 [0;2]	0 [−2;0]	**0**	0	2
**C1**	0 [−2;4]	6 [−8;−6]	2 [0,2]	2 [2;2]	0 *[0;3]*	**2**	0	2
**C2**	0 [−2;2]	0 [−2;0]	0 [−4;2]	0 [0;4]	2 [−4;0]	**0**	0	0
**D1**	4 [1;8]	16 [−22;−6]	0 [−2;2]	2 [−2;6]	2 [−4;0]	**2**	2	4
**D2**	2 [−2;8]	2 [−4:−2]	0 [0,2]	4 [−6;−2]	*0* [0;3]	**2**	0	2
**D3**	10 [8;18]	8 [−8;−6]	0 [−2;1]	0 *[−2;2]*	*2* *[0;4]*	**2**	0	8

**Table A4:** Users’ number of steps to reach the cadence of 100 steps/min.

	USER 1	USER 2	USER 3	USER 4	USER 5	Median	Q1	Q3
***REF***	-	-	-	-	-	**-**	-	-
**A1**	7	30	30	20	10	**20**	10	30
**A2**	1	30	7	6	30	**7**	6	30
**A3**	nc	30	30	30	1	**30**	22.75	30
**A4**	23	30	12	1	16	**16**	12	23
**A5**	30	1	7	15	25	**15**	7	25
**A6**	30	9	30	5	20	**20**	9	30
**B1**	6	30	1	9	6	**6**	6	9
**B2**	9	30	6	1	1	**6**	1	9
**B3**	30	14	1	1	1	**1**	1	14
**B4**	error	30	1	1	1	**1**	1	8.25
**B5**	30	6	2	1	1	**2**	1	6
**B6**	30	1	1	1	1	**1**	1	1
**C1**	7	30	3	1	6	**6**	3	7
**C2**	4	1	6	1	6	**4**	1	6
**D1**	17	30	1	7	5	**7**	5	17
**D2**	10	1	4	30	6	**6**	4	10

**Table A5:** Users’ cadence variability.

	USER 1	USER 2	USER 3	USER 4	USER 5	Median	Q1	Q3
***REF***	*1.97*	*2.80*	*2.61*	*1.64*	*3.77*	***2.61***	*1.97*	*2.80*
**A1**	2.99	1.35	5.41	2.28	1.71	**2.28**	1.71	2.99
**A2**	3.23	−0.40	−0.08	4.67	5.16	**3.23**	−0.08	4.67
**A3**	NaN	2.41	2.81	3.81	1.22	**2.61**	2.11	3.06
**A4**	4.86	3.78	6.44	2.42	1.73	**3.78**	2.42	4.86
**A5**	0.55	1.11	1.75	5.01	23,20	**1.75**	1.11	5.01
**A6**	2.68	−0.10	15,49	0.71	8.47	**2.68**	0.71	8.47
**B1**	2.04	−0.52	−0.33	1.26	0.25	**0.25**	−0.33	1.26
**B2**	2.40	1.29	4.69	0.64	−1.56	**1.29**	0.64	2.40
**B3**	−0.24	0.16	−0.11	0.49	−1.46	**−0.11**	−0.24	0.16
**B4**	NaN	1.04	2.75	0.82	−1.50	**0.93**	0.24	1.47
**B5**	0.07	−0.90	−0.43	1.15	−0.18	**−0.18**	−0.43	0.07
**B6**	2.64	−0.18	−0.53	0.47	−1.36	**−0.18**	−0.53	0.47
**C1**	5.22	−0.47	1.16	−0.01	−0.87	**−0.01**	−0.47	1.16
**C2**	3.94	0.64	0.92	0.79	−0.07	**0.79**	0.64	0.92
**D1**	2.02	7.53	−0.19	8.57	−0.13	**2.02**	−0.13	7.53
**D2**	3.71	−0.61	0.77	1.78	−0.65	**0.77**	−0.61	1.78
**D3**	4.54	0.80	−0.15	1.89	−0.43	**0.80**	−0.15	1.89

**Table A6:** Rhythmic asymmetry: users’ asymmetry index on cadence.

	USER 1	USER 2	USER 3	USER 4	USER 5	Median	Q1	Q3	IQR
***REF***	0.8	0.0	1.8	0.0	2.0	**0.8**	0.0	1.8	1.8
**A1**	2.0	1.1	0.0	1.0	0.0	**1.0**	0.0	1.1	1.1
**A2**	0.0	0.0	0.0	1.0	0.0	**0.0**	0.0	0.0	0.0
**A3**	NaN	0.0	0.0	0.0	0.0	**0.0**	0.0	0.0	0.0
**A4**	1.9	0.0	0.0	0.0	1.0	**0.0**	0.0	1.0	1.0
**A5**	0.0	1.0	2.0	0.0	2.0	**1.0**	0.0	2.0	2.0
**A6**	0.0	0.0	4.0	0.0	2.0	**0.0**	0.0	2.0	2.0
**B1**	2.0	1.1	0.0	1.0	1.0	**1.0**	1.0	1.1	0.1
**B2**	0.0	0.0	0.0	1.0	1.0	**0.0**	0.0	1.0	1.0
**B3**	0.0	1.1	2.0	0.0	0.0	**0.0**	0.0	1.1	1.1
**B4**	NaN	0.0	0.0	0.0	0.0	**0.0**	0.0	0.0	0.0
**B5**	0.0	2.1	2.0	0.0	0.0	**0.0**	0.0	2.0	2.0
**B6**	0.0	0.0	1.0	1.0	0.0	**0.0**	0.0	1.0	1.0
**C1**	0.0	1.1	0.0	0.0	0.0	**0.0**	0.0	0.0	0.0
**C2**	0.0	0.0	1.0	0.0	1.0	**0.0**	0.0	1.0	1.0
**D1**	1.0	0.0	0.0	0.0	0.0	**0.0**	0.0	0.0	0.0
**D2**	2.0	0.0	0.0	2.1	1.0	**1.0**	0.0	2.0	2.0
**D3**	0.9	1.1	1.0	1.0	0.0	**1.0**	0.9	1.0	0.1

**Table A7:** Spatial asymmetry: Users’ asymmetry in stride length.

	USER 1	USER 2	USER 3	USER 4	USER 5	Median	Q1	Q3
***REF***	*2.1*	*0.3*	*5.1*	*0.0*	*1.7*	***1.7***	*0.3*	*2.1*
**A1**	0.2	2.6	18,9	3.9	0.0	**2.6**	0.2	3.9
**A2**	−1.1	1.6	−4.1	0.1	1.7	**0.1**	−1.1	1.6
**A3**	NaN	6.2	−2.5	2.3	0.1	**1.2**	−0.6	3.3
**A4**	1.4	2.7	−1.0	1.5	−0.1	**1.4**	−0.1	1.5
**A5**	−0.1	2.0	−3.2	2.3	−1.1	**−0.1**	−1.1	2.0
**A6**	0.1	0.3	−2.6	0.6	−1.7	**0.1**	−1.7	0.3
**B1**	4.0	3.5	−3.6	0.3	0.1	**0.3**	0.1	3.5
**B2**	1.0	2.7	−4.8	2.3	−1.6	**1.0**	−1.6	2.3
**B3**	0.0	1.5	−1.1	4.8	−1.3	**0.0**	−1.1	1.5
**B4**	NaN	−0.2	−2.6	3.8	−1.3	**−0.8**	−1.6	0.8
**B5**	−1.6	1.8	−2.9	1.3	2.6	**1.3**	−1.6	1.8
**B6**	0.6	0.8	−3.7	3.4	1.0	**0.8**	0.6	1.0
**C1**	1.2	0.5	−2.6	0.5	0.3	**0.5**	0.3	0.5
**C2**	2.1	6.3	−5.0	2.5	5.8	**2.5**	2.1	5.8
**D1**	−0.3	0.9	−3.5	0.9	1.7	**0.9**	−0.3	0.9
**D2**	0.0	2.1	−4.4	1.9	0.8	**0.8**	0.0	1.9
**D3**	1.2	4.8	−2.5	0.0	−1.5	**0.0**	−1.5	1.2
